# Real-Life GOLD 2011 Implementation: The Management of COPD Lacks Correct Classification and Adequate Treatment

**DOI:** 10.1371/journal.pone.0111078

**Published:** 2014-11-07

**Authors:** Vladimir Koblizek, Ladislav Pecen, Jaromir Zatloukal, Jana Kocianova, Marek Plutinsky, Vitezslav Kolek, Barbora Novotna, Eva Kocova, Sarka Pracharova, Ales Tichopad

**Affiliations:** 1 Pulmonary Department, Faculty of Medicine in Hradec Kralove, Charles University in Prague and University Hospital Hradec Kralove, Hradec Kralove, Czech Republic; 2 CEEOR Institute, Prague, Czech Republic; 3 Department of Respiratory Medicine and Tuberculosis, Faculty of Medicine and Dentistry, Palacky University Olomouc and University Hospital Olomouc, Ostrava Poruba, Czech Republic; 4 Chest Clinic, Ostrava Poruba, Czech Republic; 5 Department of Respiratory Medicine and Tuberculosis, Faculty of Medicine, Masaryk University Brno and University Hospital Brno, Brno, Czech Republic; University of Dundee, United Kingdom

## Abstract

Chronic obstructive pulmonary disease (COPD) is a serious, yet preventable and treatable, disease. The success of its treatment relies largely on the proper implementation of recommendations, such as the recently released Global Strategy for Diagnosis, Management, and Prevention of COPD (GOLD 2011, of late December 2011). The primary objective of this study was to examine the extent to which GOLD 2011 is being used correctly among Czech respiratory specialists, in particular with regard to the correct classification of patients. The secondary objective was to explore what effect an erroneous classification has on inadequate use of inhaled corticosteroids (ICS). In order to achieve these goals, a multi-center, cross-sectional study was conducted, consisting of a general questionnaire and patient-specific forms. A subjective classification into the GOLD 2011 categories was examined, and then compared with the objectively computed one. Based on 1,355 patient forms, a discrepancy between the subjective and objective classifications was found in 32.8% of cases. The most common reason for incorrect classification was an error in the assessment of symptoms, which resulted in underestimation in 23.9% of cases, and overestimation in 8.9% of the patients' records examined. The specialists seeing more than 120 patients per month were most likely to misclassify their condition, and were found to have done so in 36.7% of all patients seen. While examining the subjectively driven ICS prescription, it was found that 19.5% of patients received ICS not according to guideline recommendations, while in 12.2% of cases the ICS were omitted, contrary to guideline recommendations. Furthermore, with consideration to the objectively-computed classification, it was discovered that 15.4% of patients received ICS unnecessarily, whereas in 15.8% of cases, ICS were erroneously omitted. It was therefore concluded that Czech specialists tend either to under-prescribe or overuse inhaled corticosteroids.

## Background

Chronic obstructive pulmonary disease (COPD) is a serious, though preventable and treatable, disease [1]–[3]. According to the World Health Organization (WHO), there are about 65 million people suffering from moderate to severe COPD. In 2005, more than three million people died of COPD, which equates to 5% of all deaths globally. COPD has recently become the third most common cause of death worldwide. Overall, COPD mortality in the 28 countries of the European Union (EU) is about 150,000 deaths per 260 million EU adults aged ≥40 years [4]–[6]. The mortality rate and socioeconomic burden are also increasing in the post-communist countries of Central Europe [7], [8]. COPD is still underestimated and underdiagnosed in the global population [9]. In addition to the global treatment strategies and guidelines, there are also several sets of national diagnostic and therapeutic recommendations [10]–[16]. The comprehensive Czech COPD guidelines are based on two elementary principles [17]. The first is based on the current GOLD strategy, and the second represents a phenotypic approach to symptomatic COPD patients (especially for subjects coming from B and D GOLD categories), similar to the Spanish COPD guidelines (GesEPOC) [12], [16]. Multiple different sets of guidelines are adopted and adhered to by doctors in various countries, and to different extents. Numerous studies point to the substantial gaps between what is recommended for COPD management, and what is actually put into practice [9], [18]–[21]. It was shown that the implementation of established guidelines was often not optimal, particularly in COPD subjects that died during the follow-up treatment [22]. Moreover, such non-adherence to the COPD guidelines may lead to an excess of direct medication costs (€59,000,000/year in Spain, for example] [23]. The adequate application of the multidimensional GOLD 2011 classification facilitates the identification of highly symptomatic patients at elevated risk of COPD, thus enabling a more personalized and adequate therapeutic approach [24]. The accuracy of GOLD 2011 as applied by practitioners in reality, however, remains a challenging issue. Globally, long-acting inhaled drugs are considered to be the prime choice for COPD pharmacotherapy [25]. GOLD 2011 proposes the optimal position of inhaled corticosteroids (ICS) in COPD maintenance therapy [24]. Due to relative novelty and complexity of GOLD 2011, as well as its overlap with other guidelines, we assume some degree of misclassification, as well as other inadequacies that may affect the proper use of ICS in COPD management.

Understanding the links between the proper implementation of GOLD 2011, patient classification and treatment patterns is expected to improve COPD management substantially, on the national level. The primary objective of this study, therefore, was to examine how correctly GOLD 2011 is being used among Czech respiratory specialists, in particular with regard to the correct classification of patients. We later explored what effect a potential misclassification could have on the use of medication contrary to guideline recommendations, in particular on the use of ICS. ICS, in particular, were considered the most critical pharmacotherapeutic factors in subgroups, such as non-exacerbating patients in A and B GOLD categories, due to their cost, their potential to produce adverse events, and a lack of evidence as to their positive effect [15], [17], [26]–[28].

## Methods

### Objectives

The scope of this paper was largely driven by findings obtained by means of explorative data analysis, pertinent, *inter alia*, to the impact of various parameters on the misclassification of patients.

### Study design

The study was designed as a multi-center, cross-sectional, observational study of COPD treatment practice among Czech respiratory specialists treating COPD, hereinafter referred to as ‘COPD specialists’. The study consisted of two types of survey, conducted via the Internet: the electronic general form (e-GF) and the patient-specific electronic case report form (e-CRF). The goal of the e-GF was to characterize each COPD specialist by collecting information about: his/her involvement in the COPD treatment; the number of COPD patients treated monthly; his/her subjective view on the diagnostic importance of selected COPD symptoms; their real, day-to-day usage of any of the COPD guidelines. The e-CRF collected information about individual patients' diagnosis, the method of diagnostics used and the therapeutic approach adopted. Both forms were administered online, using a remote data capture system for specialists operated by CEEOR, a contract research organization responsible for contacting the COPD specialists, and for data collection and analysis. The study was further backed and promoted by the Czech Pneumological and Phthisiological Society (CPPS).

Two hundred COPD specialists treating outpatients—members of the CPPS with previous experience in various types of surveys run by CEEOR—were initially contacted via email, sent on behalf of the CPPS by the CEEOR team. For the purpose of further analysis, they were classified with regard to their medical facility as: I) those working in an outpatient, ambulatory setting; II) those working in an ambulatory care unit within a hospital facility and; III) those working in ambulatory care in a tertiary or university hospital. Following their recruitment, COPD specialists were provided with individual access codes, and were instructed to visit the homepage of the CPPS, where the e-GFs and the e-CRFs were available via prominent Internet links. The website also contained a brochure describing the study's course and design, as well as the role expected of the COPD specialist. COPD specialists were requested to complete the e-GFs first and then, within one week, to enter the clinical, functional and medical history parameters of ten consecutive COPD patients into the e-CRFs. No patient was allowed to be omitted, unless meeting the single exclusion criterion—acute exacerbation of COPD. For the sake of data management and validation, each patient record was assigned a unique, random ID, generated by the e-CRF. The ID was recorded in parallel by the COPD specialist into the respective patient's chart, in order to facilitate later validation of records. COPD specialists were discouraged from tracking the patients in the e-CRFs in any other way which could facilitate the disclosure of a patient's identity.

The study was non-interventional, and the patients' personal data remained undisclosed throughout the course of the study. No drug or medical device's safety and effectiveness were studied in particular. In accordance with the laws of the Czech Republic, the study was thus not the subject of an ethical committee approval, and neither was informed consent required to be obtained from the patients. The study was reported to the State Institute of Drug Control (SUKL) and, following its completion, a copy of the final report was also submitted.

### Inclusion and classification of patients

COPD specialists were requested to trace all their patients, both those diagnosed with COPD in the past and those seeing them currently, with the exception of those who consulted their COPD specialist with acute exacerbation of COPD. In addition, patients with asthma, or overlap of asthma and COPD, were excluded. The time window for the patients' enrolment was seven days from the day on which the e-GF survey was completed.

Besides recording the primary clinical parameters - the bronchial obstruction, number of acute exacerbations, modified Medical Research Council (mMRC) dyspnea scale and COPD Assessment Test (CAT)—COPD specialists were also instructed to classify each patient into one of the categories, A, B, C, or D, as proposed in GOLD 2011. This classification is herein considered the ‘subjective classification’ [12]. By contrast, the ‘objective classification’ was rigorously derived by applying the GOLD 2011 algorithm, using software (SAS 9.3 for MS Windows), to the primary clinical parameters [12]. In cases where both mMRC and CAT were available in a patient, the worst of the two was used. CAT is a multidimensional tool comprising dyspnea, among other symptoms. For this reason, it is preferred over the mMRC [12].

The use of ICS was, in accordance with GOLD 2011, considered adequate in the cases of patients categorized into C and D groups. The use of ICS in categories A and B was considered to be a deviation from guideline recommendations.

### Statistical analysis

All data were statistically described using SAS 9.3 for MS Windows. The expected objective classification was compared with the observed subjective classification by means of absolute and relative frequencies, and the discrepancy was tested by the Kappa test, using α = 0.05. The effect of the doctor's gender, patient's age, district, type of medical facility, and number of COPD patients treated monthly, on the error frequency in diagnosis was determined by relative frequencies, and tested by the Chi-square test, using α = 0.05. Similarly, the same factors were described and tested for their effect on the failure to prescribe ICS in line with guideline recommendations. The effect of primary parameters, such as bronchial obstruction, number of acute exacerbations, modified mMRC dyspnea scale, and CAT, on the error rate of the GOLD 2011 classification was also described by relative frequencies, and presented, both in terms of an isolated effect and in concurrence with the alternative parameter, on the same axis [12], [29]. Missing data were handled as they were; no imputation technique was used. To evaluate how an individual CAT contributed to an erroneous classification, only non-missing entries were considered.

## Results

### Characterization of COPD specialists

**Figure 1:**
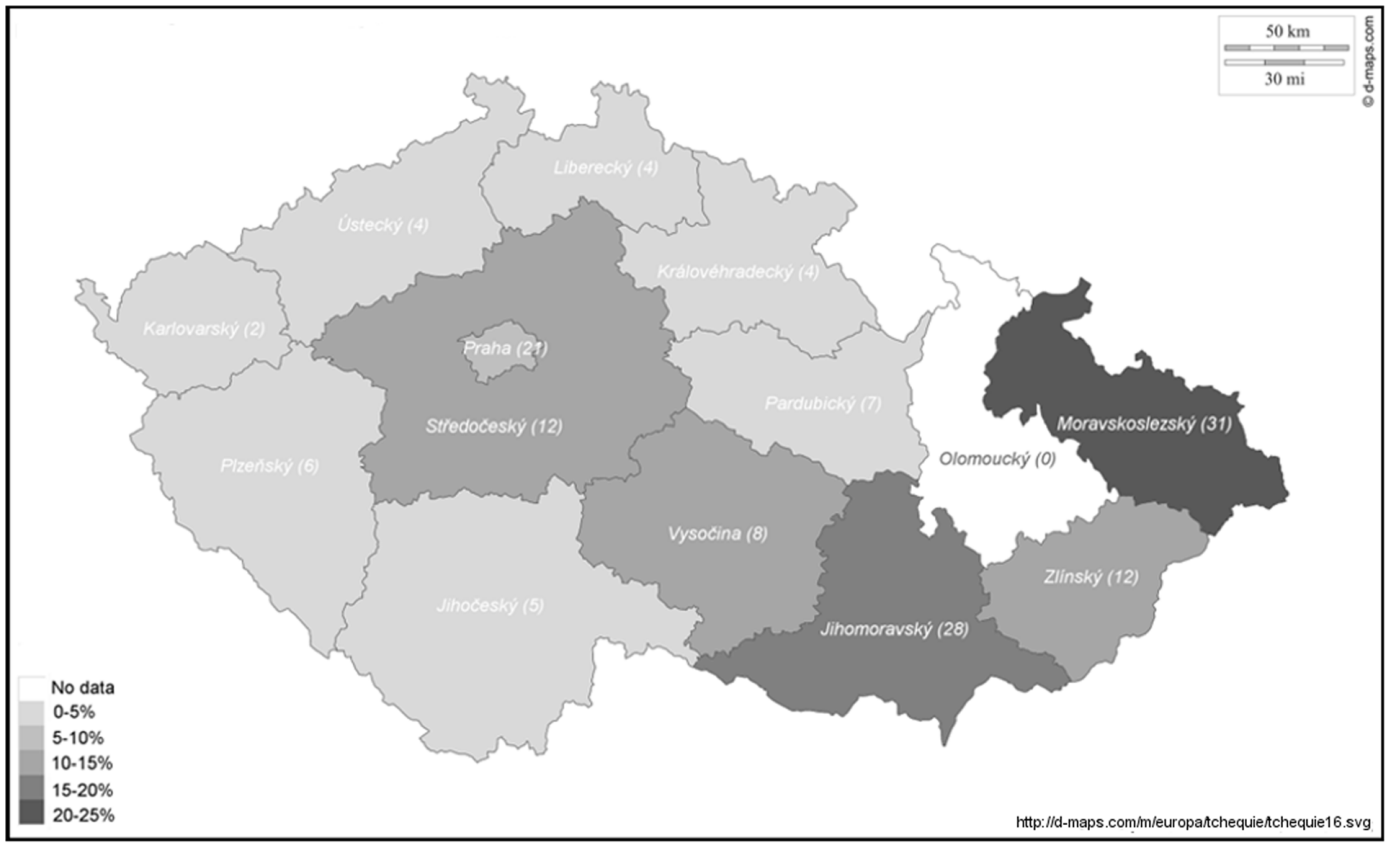
Numbers of COPD specialists involved in the research by country regions of the Czech Republic.

**Figure 2:**
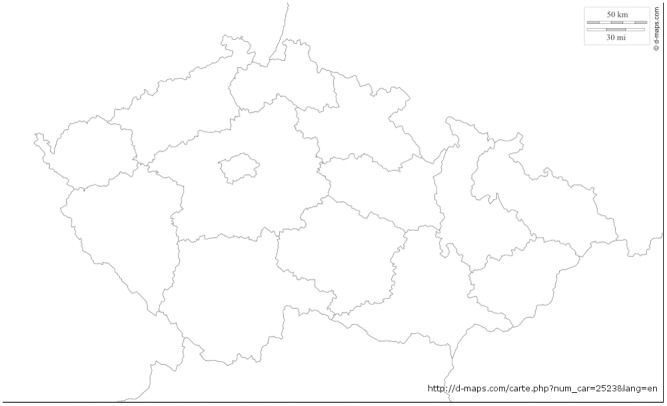
Original version of the Czech Republic map in SVG format.

The study was conducted from October 2nd 2012 to December 2nd 2012. In total, the data from 144 COPD specialists were obtained, 80 of which (55.6%) worked in an outpatient ambulatory care setting, 19 (13.2%) in an ambulatory care unit within a hospital facility, and 45 (31.2%) in ambulatory care of a tertiary or university hospital, as indicated in the e-GFs. Varied numbers of COPD specialists were involved in the 14 regions of the Czech Republic, ranging from 0 (one region only) to 31 (Figure 1, original version of the Czech Republic map in SVG format is attached as Figure 2). Symmetric portions of hospital-based and office-based COPD specialists (44.4% vs. 55.6%) participated in our study.

In total, 143 (99.3%) of all COPD specialists involved indicated GOLD 2011 as the leading set of guidelines in their practice, often alongside other recommendations, most frequently the “previous version” of the Czech guidelines (83.3%), but also the American College of Physicians, American College of Chest Physicians, American Thoracic Society, and European Respiratory Society (ACP/ACCP/ATS/ERS) guideline update (50.7%), the International Primary Care Respiratory Group (IPCRG) - COPD guidelines (10.4%), the National Institute for Health and Care Excellence (NICE) guidelines (8.3%), and other sources (4.9%). COPD specialists estimated the number of consultations as ranging from 10 to 500 patients per month. With regard to the importance of selected symptoms for clinical diagnostics of COPD, almost all physicians (97.9%) considered the shortness of breath and persistent and productive cough (96.5%) as the principal symptoms of COPD. Accompanying symptoms, such as sputum production (92.4%), exposures to environmental inhaled risk factors (83.3%), wheezing and chest tightness (81.3%), and frequent respiratory infections (79.2%), were reported to provide additional advantage in diagnostics of COPD.

### Characterization and classification of COPD patients

In total, 1,355 e-CRFs of COPD patients were provided, 512 (37.8%) of which were from males and 843 (62.2%) were from females. 129 COPD specialists provided the data of 10 patients as requested, while 15 provided the data of fewer than 10 patients. The average age of the patients was 68.5 (MEDIAN 69, SD 9.66) years, the average mMRC was 1.807 (MEDIAN 2.000, SD 1.126), the average CAT was 18.343 (MEDIAN 18.0, SD 9.068), and the average number of exacerbations was 0.754 (MEDIAN 1.0, SD 0.802). The average bronchial severity, based on the post bronchodilator FEV_1_, was GOLD 1 (10.3%), GOLD 2 (46.0%), GOLD 3 (30.6%) and GOLD 4 (13.1%). Regarding the GOLD 2011 classification, COPD specialists subjectively classified 356 patients into A, 427 into B, 229 into C and 343 into D category (Table 1). However, applying the objectively computed rigorous classification rules to the primary clinical parameters, there ought to have been 385 patients in group A, 304 in group B, 74 in group C and 592 in group D. Hence, there was a significant discrepancy (Kappa test, p<0.0001) between the subjective and objective classifications, indicating misclassification in 32.8% of all reported cases.

**Table 1:** Comparison between subjective classification as reported by COPD specialists and objective software-computed classifications based on GOLD 2011 rules for individual patients.

Objective classification by GOLD 2011 rule	Subjective classification by doctor				
	←Under-/Over-classification→				
	A	B	C	D	Total
					
A	303	75	7	0	385
	22.36%	5.54%	0.52%	0%	28.41%
B	42	230	26	6	304
	3.1%	16.97%	1.92%	0.44%	22.44%
C	7	14	47	6	74
	0.52%	1.03%	3.47%	0.44%	5.46%
D	4	108	149	331	592
	0.3%	7.97%	11%	24.43%	43.69%
Total	356	427	229	343	1355
	26.27%	31.51%	16.9%	25.31%	100%

The top number represents the absolute number of COPD subjects and the bottom number indicates relative (%) frequency out of the entire sample of patients.

On closer examination, 23.9% of all cases were underestimated and 8.9% of all cases were overestimated. In those underestimated, the three most frequent errors were as follows: D was misclassified as C in 25.2% of cases. D was also misclassified as B in 18.2%. B was misclassified as A in 13.8%. 44.1% of all correctly classified D cases were underestimated, 28.4% of all correctly classified C cases were underestimated and 13.8% of all correctly classified B cases were underestimated. In those overestimated, A was misclassified as B in 19.5%. B was misclassified as C in 8.6%, and C was misclassified as D in 8.1%. 21.3% of all correctly classified A cases were overestimated, 10.5% of all correctly classified B cases were overestimated, and 8.1% of all correctly classified C cases were overestimated.

**Figure 3:**
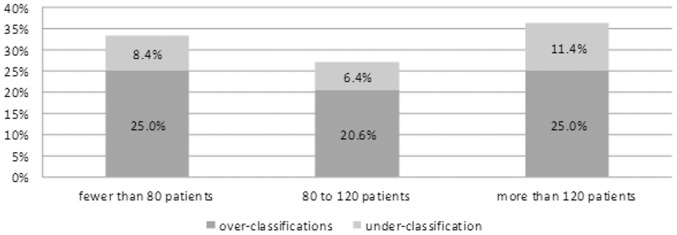
Effect of number of COPD patients seen in a month on a discrepancy between subjectively classified patients into GOLD 2011 groups and classification achieved by objective assessment using software.

**Figure 4:**
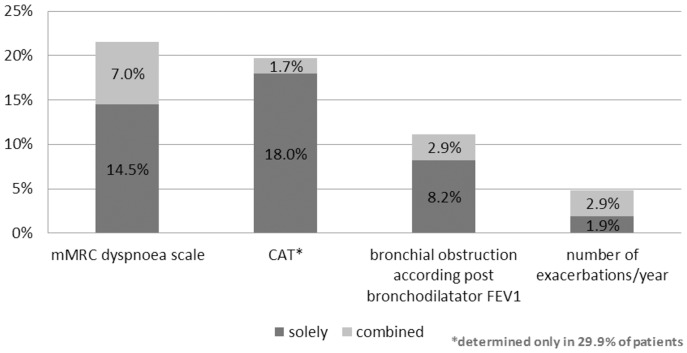
Contribution to the misclassification of an individual clinical component interpreted within the GOLD 2011 classification matrix. The percentage indicates relative frequency of misclassified cases attributable to a given clinical component. A sole effect is responsible for misclassified cases due to an obvious error in interpretation of one specific primary clinical parameter. The combined effect is responsible for misclassified cases due to an obvious error in interpreting both clinical criteria on the same axis (mMRC dyspnea scale and CAT respective bronchial obstruction and number of COPD exacerbations/year). *Statistical probabilities were calculated of pair-wise contrasts as follows; mMRC dyspnea scale vs. CAT (p>0.05), mMRC dyspnea scale vs. bronchial obstruction based on post bronchodilator FEV1 (p<0.0001), mMRC dyspnea scale vs. number of exacerbations/year (p<0.0001), CAT vs. bronchial obstruction (p<0.0001), CAT vs. number of exacerbations/year (p<0.0001), bronchial obstruction vs. number of exacerbations/year (p<0.0001).*

As for the factors associated with the misclassification, the number of COPD patients treated monthly showed to significantly influence the frequency of misclassified cases. The greatest error rate (36.7%) was observed in specialists with more than 120 COPD patients per month, followed by the specialists with fewer than 80 patients per month (33.4%). The smallest error rate was in the middle group with 80 to 120 patients per month (27.0%). The difference was statistically significant, with p = 0.0273. Specifically, underestimation occurred in the group with more than 120 patients per month in 25.3% of cases, in the group with fewer than 80 patients per month in 25.0% of cases, and in the group with 80 to 120 patients per month in 20.6% of cases. Overestimation occurred in the group with more than 120 patients per month in 11.4% of cases, in the group with fewer than 80 patients per month in 8.4% of cases, and in the group with 80 to 120 patients per month in 6.4% of cases (Figure 3). Specialist's gender, patient's age, and district and type of medical facility were found to have no significant effect. The effect of clinical criteria, such as the bronchial obstruction (post bronchodilator FEV_1_), the number of exacerbations per year, mMRC dyspnea scale and CAT, on the error rate of the GOLD 2011 classification was described by relative frequencies, and presented both in terms of an isolated effect and in concurrence with the alternative parameter, on the same axis of the classification matrix (Figure 4). The most common reason for the GOLD misclassification was an incorrect evaluation of symptoms (21.5% in mMRC dyspnea scale and 19.7% where CAT was done), followed by bronchial obstruction according to post bronchodilator FEV_1_ 11.1%, whereas the lowest impact on proper classification (5.8%) was made by the number of exacerbations per year.

### Adequacy of maintenance medication

**Figure 5:**
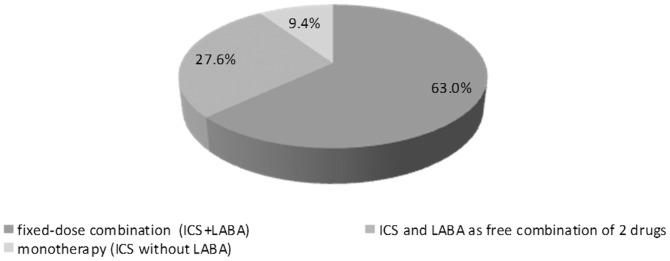
Use of ICS in monotherapy or combination therapy.

Patients were treated, in monotherapy or combination, with long-acting beta agonist (LABA, 74.2%), followed by ICS (49.5%), long-acting muscarinic antagonists (LAMA, 45.9%) and roflumilast (4.80%). Combination LABA+ICS was used in 44.9% of cases, ICS monotherapy only in 4.7% (9.4% of patients with ICS). Fixed-dose combination of ICS and LABA was used in 31.2% (63.0% of patients with ICS), while free combination of these two drugs was used in 13.7% of patients (27.6% of patients with ICS) (Figure 5).

**Figure 6:**
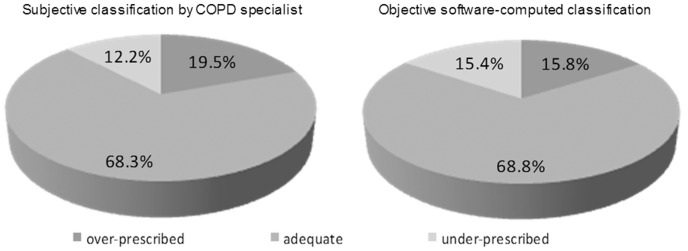
Comparison of ICS prescription related to the GOLD 2011 classification as done subjectively by COPD specialist and as objectively computed.

Even while being guided by the specialists' subjective classification, 19.5% of patients were still over-prescribed with ICS, whereas 12.2% of them were under-prescribed with ICS (Figure 6). On the other hand, it was discovered that, as regards the objective classification, 15.4% of ICS were prescribed unnecessarily while, in 15.8% of cases, the ICS were not prescribed despite the fact that such a prescription would have been appropriate given the patient's condition (Figure 6).

Women prescribed contrary to guideline recommendations more frequently than men (33.6% vs. 27.3%, p = 0.042), predominantly by over-prescribing ICS.

Furthermore, there was a significant effect caused by the type of medical facility (p = 0.0022). Over-prescription in specialists working in ambulatory care outside a hospital was present in 18.1% of cases at this type of facility, as compared with the ambulatory care in a tertiary or university hospital, where only 8.7% of cases were over-prescribed ICS. Conversely, the same two types of facilities showed 13.1% and 21.9% of cases with under-prescription of ICS, respectively. It was found, however, that the number of patients seen per month had no significant effect, and that, interestingly, the smallest failure rate was in those specialists seeing fewer than 80 patients per month (28.7%), among whom under-prescription was most common, while the highest failure rate (34.6%) came from those specialists seeing more than 120 patients per month, among whom over-prescription was most common.

## Discussion

We discovered a satisfactory, self-reported acceptance of the international and local guidelines in the field of COPD among the Czech specialists. Nevertheless, the real-life implementation of these instruments was found to be insufficient. We observed a systematic GOLD 2011 under-classification, while patients were simultaneously being over-treated with ICS. The greatest classification error was present among doctors treating large numbers of COPD patients monthly. The most prominent driver responsible for GOLD misclassification was the incorrect evaluation of symptoms, while evaluation of exacerbation frequency contributed to errors the least. The GOLD 2011 classification, applied objectively, showed a predominance of highly symptomatic patients classified either as B or D (jointly 66.1%) and a rather rare incidence (5.5%) of a few C patients, with few symptoms, suffering from severe bronchial obstruction and/or frequent exacerbations. Most alarming are those 19.5% of patients for whom ICS were over-prescribed, and those 12.2% for whom it was under-prescribed. Women prescribed contrary to guideline recommendations more frequently than men, mainly by over-prescribing ICS.

The best available evidence-based guidelines can improve the positive clinical impact of COPD management [30]. Identifying the target audience, deciding what type of evidence to include, and establishing, reporting and publishing guidelines are only the first steps. Educational activity, as well as disseminating, implementing, evaluating and updating these guideline documents, are the necessary next steps toward a real, everyday medical application [30]–[34]. In parallel to the worldwide extended ACP/ACCP/ATS/ERS diagnosis and COPD treatment guidelines, which provide an up-to-date and uniform COPD management strategy, we can also use several local COPD recommendations from Canada, the United Kingdom, Spain, India and the Czech Republic [11]–[17]. This observational study showed relatively broad preferences among Czech COPD specialists, ranging from the current version of GOLD, via the Czech COPD guidelines, to ACP/ACCP/ATS/ERS and NICE guidelines. A closer look at real-life COPD management is, therefore, particularly interesting. The popularity and self-declared knowledge of the GOLD 2011 strategy, as reported in our study, was extremely high (more than 99% of physicians), a finding that corresponds with the works of other authors. Excellent knowledge of the current version of the GOLD recommendations among respiratory specialists and primary care physicians in Germany were repeatedly described by Glaab [18], [19]. A high level of knowledge of GOLD among respiratory specialists, and sufficient knowledge among family physicians and internists, was also demonstrated in Nigeria [20].

It is not unusual for a certain amount of error to be introduced into large surveys, mainly through insufficient surveillance of the potential biases in the selection strategy adopted in the recruitment of the ‘specialists’ and ‘patients’ subpopulations, whether determined geographically, demographically, by severity of disease, or otherwise. The vast majority of COPD patients in Central and Eastern Europe (CEE) are treated by specialized respiratory specialists [17]. It was for this reason that our project targeted this group - specialists - rather than general practitioners or internists [20], [21], [35]. In the Czech Republic, there were 371 respiratory specialists providing specialized outpatient health care in 2012 [36], of which 144 confirmed their involvement in COPD treatment and were willing to participate in our study (38.8%). These COPD specialists were a good reflection of the population distribution between hospital-based and office-based doctors. The official national figure for the number of patients diagnosed with, and monitored for, COPD reached 214,978 in 2012, representing 2,044 COPD cases per 100,000 residents of the Czech Republic [36]. Thus, the 1,355 COPD patients enrolled on the study constitute more than 0.6% of the Czech COPD population. To verify the sample's representativeness, we could confirm that the spectrum of bronchial obstruction severity (GOLD 1, 2, 3, 4) among the patients in the study closely resembled that of the COPD population, as officially reported [36]. As in a study recently conducted in Italy, the majority of patients in our study suffered from moderate COPD (46% vs. 51.5% in Italy), and belonged to the D category (43.7% objectively classified patients vs. 45.6% in Italy) [37].

It was demonstrated that the number of patients under COPD specialists' care varies substantially depending on the facility type, with university and tertiary hospitals treating a rather smaller number of patients per month than outpatient clinics. The apparent consistency of the main symptoms leading to the diagnosis of COPD (breathlessness in the first instance) is in line with the generally acknowledged diagnosis criteria, and is comparable to what has been found elsewhere [9].

As a primary finding, one third of the patients were incorrectly classified in our study. Misclassification appeared in all three types of medical facility, irrespective of the specialist's gender, patient's age, or region. The risk of erroneous classifications, whether over- or under-classifications, is associated with an increase in the number of patients seen. This may indicate that a routine subjective assessment is often preferred, and used instead of an accurate algorithm. Similarly, we presume that time-consuming, yet crucially important, indicators, such as the mMRC and the CAT, may be assessed inconsistently, erratically or erroneously, thus introducing further risk of error into the classification process. This could be supported by this study's findings on mMRC and CAT, both having been found statistically, and significantly, to be associated with a similar percentage of misclassified patients (mMRC 21.5% and CAT 19.7%). Rather surprisingly, the assessed incidence of acute exacerbations was only weakly associated with misclassification.

The over-prescription of ICS—that is, their use in patients in A and B groups, as subjectively assessed by the COPD specialists—was observed rather frequently, in almost 20% of all patients. It was more common among physicians seeing more than 120 patients per month. Similar to our findings, Sarc et al. reported that ICS over-prescription reached 25% [22] and presented in relation to GOLD 2006 disease stages I and II. Overtreatment with ICS has also been reported elsewhere [38]–[40]. Considering the objective classification, as derived by computer software based on the rigorous GOLD 2011 rules and primary clinical parameters, we observed over-prescription in 15.4% of cases, as well as under-prescription in 15.8% of cases, thus proving a highly symmetric inappropriate use of ICS. It is interesting that the rate of correct indication of ICS is almost identical (statistically non-significant difference) in the case of the two classification schemes: GOLD 2011 (ICS for categories C and D) - correct indication in 68.78% and GOLD 2006 (recommending ICS for III.- IV. stadium) - correct classification in 67.60%.

Regarding trends in the prescription of inhaled medications, our study shows that only 45.9% of patients with COPD used LAMA medication. Inadequate use of ICS in low-risk patients, as determined by the GOLD categories (non-C and non-D), was also indicated within the Adelphi Respiratory Disease Specific Programme [24]. The situation was also similar in the USA, where use of ICS was reported, increasingly, to predominate [41]. Unlike in the above-mentioned studies, the Belgian COPD Working Group discovered that, as regards the management of the more severe COPD stages, LAMA were the predominantly used medications, administered in 79% of cases, as compared to LABA, which were used in 36% to 48% of cases, or to ICS, which were used in 21% to 67% of cases [9]. Where a combination of LABA ICS was prescribed to patients, free dose combination was used in 25% of cases. The reason for this is that a free combination was cheaper than a fixed combination of products available on the Czech market.

Based on our results, it seems that half of the patients (49.2%) are classified correctly and treated according to guideline recommendations. Almost 20% of patients are treated correctly despite erroneous classification, with 14.2% being under-classified and 5.5% over-classified. On the other hand, out of the 18.1% patients with correct classification, 36% weren't prescribed ICS, while the rest received ICS contrary to guideline recommendations.

Concordant error—that is, under-classification and under-prescription—was found in 8.6% of cases, while over-classification and over-prescription was observed in only 3.1% of our cases. The combination of under-classification with over-prescription, or over-classification with under-prescription, was rarely seen, occurring in only 1.2% and 0.3% of cases, respectively. Finally, a relatively small number of COPD subjects were treated badly with poor classification—this happened in 11.7% (8.6%+3.1%) of all consecutive real-life cases.

Appropriate and adequate diagnostics and treatment adjusted to disease severity is the key to good disease management, resulting not only in a stabilized, or even improved, condition of health and chance of survival [22], but also in substantial cost savings [23]. Hence, the effort to translate the COPD evidence-based guidelines into clinical reality is not only a theoretical goal, but also an objective of studies in Europe, including CEE [21], [42], [43].

The study's strengths lie in the high participation of healthcare practitioners and generally good regional representativeness, covering close to 40% of the relevant specialists' population in 13 out of the 14 regions involved. Furthermore, COPD was diagnosed with high certainty by experienced pulmonary specialists.

As to the potential limitations of this study, although all participating physicians conducted post-bronchodilator flow-volume loop, the absolute values of spirometric parameters were not captured and analyzed. Only the GOLD 1, 2, 3, 4 categories of bronchial obstruction were recorded, which meant that some important therapeutic modalities, such as the pulmonary rehabilitation and vaccination, were not studied. The timing of the study between October and November could, theoretically, have caused a systematic exclusion of certain types of COPD patients manifesting exacerbations of a seasonal character. We also observed a 4∶6 male-to-female ratio in our study, which does not perfectly correspond to what is known among the overall COPD population (6∶4) [36]. We attribute this, however, solely to the randomized selection process.

## Conclusions

We conclude that, despite the high awareness of the GOLD 2011 strategy among Czech COPD specialists, its real-life implementation is rather insufficient. Every second COPD subject is incorrectly classified and/or treated. There is an obvious tendency towards under-classification of patients, and a simultaneous overtreatment of COPD with ICS, resulting in an increased risk of adverse effects and unnecessary costs. Overall, specialists' poor symptom assessment is the key driver of erroneous classification. Inadequate use of ICS was observed in one-third of patients. Our results therefore justify education targeted at pulmonologists in the Czech Republic.
